# X-ray crystal structure of a designed rigidified imaging scaffold in the ligand-free conformation

**DOI:** 10.1107/S2053230X2400414X

**Published:** 2024-05-20

**Authors:** Matthew P. Agdanowski, Roger Castells-Graells, Michael R. Sawaya, Duilio Cascio, Todd O. Yeates, Mark A. Arbing

**Affiliations:** a Department of Chemistry and Biochemistry, University of California Los Angeles, Los Angeles, CA 90095, USA; b Department of Biological Chemistry, University of California Los Angeles, Los Angeles, CA 90095, USA; c UCLA–DOE Institute for Genomics and Proteomics, University of California Los Angeles, Los Angeles, CA 90095, USA; d Howard Hughes Medical Institute, University of California Los Angeles, Los Angeles, CA 90095, USA; Centro Nacional de Biotecnología – CSIC, Spain

**Keywords:** DARPins, protein cages, protein design, imaging scaffolds

## Abstract

Imaging scaffolds composed of designed protein cages fused to designed ankyrin repeat proteins (DARPins) have enabled the structure determination of small proteins by cryogenic electron microscopy (cryo-EM). One particularly well characterized scaffold type is a symmetric tetrahedral assembly composed of 24 subunits, 12 A and 12 B, which has three cargo-binding DARPins positioned on each vertex. Here, the X-ray crystal structure of a representative tetrahedral scaffold in the apo state is reported at 3.8 Å resolution. The X-ray crystal structure complements recent cryo-EM findings on a closely related scaffold, while also suggesting potential utility for crystallographic investigations. As observed in this crystal structure, one of the three DARPins, which serve as modular adaptors for binding diverse ‘cargo’ proteins, present on each of the vertices is oriented towards a large solvent channel. The crystal lattice is unusually porous, suggesting that it may be possible to soak crystals of the scaffold with small (≤30 kDa) protein cargo ligands and subsequently determine cage–cargo structures via X-ray crystallography. The results suggest the possibility that cryo-EM scaffolds may be repurposed for structure determination by X-ray crystallography, thus extending the utility of electron-microscopy scaffold designs for alternative structural biology applications.

## Abstract 2

The structure of an imaging scaffold engineered to bind and study therapeutic protein targets has been determined at 3.8 Å resolution. Cargo protein-binding DARPins are positioned within the large solvent channels of an unusually porous crystal lattice, suggesting that it may be possible to soak crystals with small target proteins in order to determine their structures.

## 1. Introduction

Imaging scaffolds composed of protein cages fused to designed ankyrin repeat proteins (DARPins) have emerged as a powerful technology for determining high-resolution structures of small proteins using single-particle cryogenic electron microscopy (cryo-EM; Liu *et al.*, 2018 ▸, 2019 ▸; Castells-Graells *et al.*, 2023 ▸; Yeates *et al.*, 2020 ▸). Binding small (∼30 kDa) protein targets to the modular DARPin domains of large, half-megadalton, symmetric scaffolds increases the size of the target into the range amenable to single-particle cryo-EM image processing. Using this approach, recent studies have achieved near-atomic resolution for small proteins, including the oncogenic protein KRAS (Castells-Graells *et al.*, 2023 ▸) in apo and ligand-bound forms. While initial development has focused on cryo-EM, we have also pursued a parallel approach to scaffold-facilitated structure determination using X-ray crystallography.

Structure determination by X-ray crystallography is a laborious process that requires extensive screening to identify conditions that produce crystals suitable for structure determination. Experimental data from high-throughput crystallization screening facilities shows that approximately 21% of protein targets subjected to screening ultimately result in crystallographic models (Lynch *et al.*, 2023 ▸). Given this relatively low success rate, there has been a strong focus on ‘salvage’ pathways to obtain structures of proteins of interest. Successful strategies include modification of protein surface properties (for example pI, hydropathy and surface entropy) by chemical modification (Kim *et al.*, 2008 ▸) and site-directed mutagenesis (Derewenda, 2004 ▸) or the use of crystallization chaperones to promote lattice formation. The latter technique can be roughly divided into two approaches: protein fusions or complexation with noncovalently bound epitope-specific protein binders. Examples of the former approach are fusion with maltose-binding protein via flexible or rigid linkers (Waugh, 2016 ▸) or the incorporation of T4 lysozyme into loops of membrane proteins (Thorsen *et al.*, 2014 ▸) to increase the solvent-accessible surface area amenable to forming crystal contacts. Examples of the latter technique are the use of protein-specific binders such as nanobodies, Fab fragments and related derivatives to generate protein complexes that are more amenable to crystallization (Koide, 2009 ▸).

Designed ankyrin repeat proteins (DARPins), which are synthetic protein-binding proteins derived from naturally occurring protein-binding motifs, have also been used as crystallization chaperones (Mittl *et al.*, 2020 ▸) and, more recently, as ‘adapters’ to bind small proteins to imaging scaffolds for cryo-EM structure determination (Liu *et al.*, 2019 ▸, 2018 ▸; Castells-Graells *et al.*, 2023 ▸). As part of a project targeting oncogenic protein targets, we generated DARPins against the C-terminal domain of the oncogenic protein BARD1 using a yeast display system and subsequently fused the anti-BARD1 DARPins to a previously characterized tetrahedral protein cage. To investigate whether cryo-EM imaging scaffolds with rigid DARPin fusions can act as crystallization chaperones, thus extending their utility for structural studies, we determined the X-ray structure of one of these imaging scaffolds in the ligand-free state. Our X-ray crystal structure suggests that electron-microscopy scaffolds may have multiple applications in the elucidation of the structures of small proteins.

## 2. Materials and methods

### 2.1. Macromolecule production

#### 2.1.1. BARD1 expression and DARPin selection

A construct encoding the BARD1 tandem BRCT domains (amino acids 423–777) with an N-terminal SUMO fusion protein followed by a HRV 3C protease site, an AVI tag and a TEV protease site was synthesized in pET-29b (Twist Bioscience). The BARD1 BRCT construct was expressed in *Escherichia coli* BL21-Gold (DE3) cells using Terrific Broth and overnight induction at 18°C with 0.5 m*M* isopropyl β-d-1-thiogalactopyranoside (IPTG); biotinylated protein was produced *in vivo* by co-expression of BirA (Addgene plasmid #102962) and the addition of biotin (final concentration 50 µ*M*) to the medium at the time of induction (Fairhead & Howarth, 2015 ▸). The cells were harvested by centrifugation, resuspended in buffer *A* (25 m*M* Tris–HCl pH 8.0, 500 m*M* NaCl, 5% glycerol, 20 m*M* imidazole, 5 m*M* β-mercapto­ethanol) supplemented with 1 m*M* EDTA, 1 m*M* phenylmethylsulfonyl fluoride and cOmplete protease inhibitor (Roche). The cells were lysed by three passes through an Emulsiflex C-3 (Avestin) at 103 MPa and the lysate was subsequently clarified by centrifugation. The SUMO-BARD1 fusion was purified from the clarified supernatant using a 5 ml HisTrap Crude FF (Cytiva) column, eluting the bound protein with buffer *B* (buffer *A* with 300 m*M* imidazole). TEV (for the removal of all N-terminal tags) or 3C protease (for the removal of the SUMO moiety but the retention of the AVI tag and TEV protease site) was added to the eluted protein and the digestion mixture was dialyzed against 2 l buffer *A* overnight at 4°C. The following day, SDS–PAGE was used to determine that digestion was complete and the reaction mixture was subsequently loaded onto a 5 ml HisTrap column, with the flowthrough collected and further purified by size-exclusion chromatography using a Superdex 75 column (Cytiva) equilibrated with buffer *C* (25 m*M* HEPES pH 7.5, 300 m*M* NaCl, 5% glycerol, 1 m*M* dithiothreitol). Fractions containing BARD1 were pooled, concentrated, flash-frozen with liquid nitrogen and stored at −80°C.

DARPins that bind BARD1 were identified using a yeast DARPin surface-display system (Morselli *et al.*, 2024 ▸). A cell population displaying BARD1 binders was enriched using two rounds of magnetic-activated cell sorting (MACS) followed by five rounds of fluorescence-activated cell sorting (FACS) using a Bio-Rad S3 cell sorter. The selections were carried out using previously described methods (Chao *et al.*, 2006 ▸; McMahon *et al.*, 2018 ▸). Briefly, the MACS experiments were performed using Dynabeads MyOne Streptavidin T1 beads (Invitrogen), while FACS experiments used an AlexaFluor488-conjugated anti-HA monoclonal antibody (Invitrogen) to select DARPin-displaying cells; cells that bound biotinylated BARD1 were selected by alternating fluorescent anti-biotin conjugates, Streptavidin R-Phycoerythrin or NeutrAvidin Rhodamine Red-X (both from Invitrogen). The target-protein concentration was decreased in each selection round to isolate higher affinity binders, with the initial MACS experiment carried out using 1.0 µ*M* protein and the final FACS selection using 30 n*M* protein. Enriched cell populations were grown in non-inducing medium and a 50 µl cell sample was centrifuged, washed with water, lysed by the addition of an equivalent volume of 40 m*M* NaOH and heated for 45 min at 95°C. This cell lysate served as the template for PCR amplification of enriched DARPin sequences; PCR amplification was carried out using primers (DARP.pYDS.Amp.For., 5′-GATGAAGTTCGTATTCTGATGGCAAATGG-3′; DARP.pYDS.Amp.Rev., 5′-CGGTGTTTTACCAAATTTATCCTGGGC-3′) that bind conserved sequences in the N- and C-caps of the DARPin. The PCR reaction used PrimeStar GXL polymerase (Takara) with a 30 s extension and 20 amplification cycles. The PCR products were purified by gel extraction and subjected to next-generation sequencing (Genewiz, New Jersey, USA). Forward and reverse reads were merged with *NGMerge* (Gaspar, 2018 ▸) and sequence abundance and characteristics were analyzed using the *MAMETS* program (Morselli *et al.*, 2024 ▸).

The most abundant DNA sequences encoding putative anti-BARD1 DARPins were synthesized and cloned into pET-29b (Twist Bioscience) with an N-terminal His_6_ tag for expression and purification. DARPins were expressed and purified using a similar procedure as for BARD1, with the substitution of 50 m*M* Tris pH 8.0, 300 m*M* NaCl, 5% glycerol, 5 m*M* β-mercaptoethanol, 20 or 300 m*M* imidazole as the affinity-chromatography buffers and 20 m*M* Tris pH 7.5, 150 m*M* NaCl as the size-exclusion chromatography buffer. Screening for DARPins that formed a stable complex with BARD1 was performed by biolayer interferometry (BLI) and was subsequently confirmed using analytical size-exclusion chromatography (AnSEC). BLI experiments were carried out with an Octet Red 96e (Sartorius) and NTA Biosensors. The His-tagged DARPins were diluted to 25 µg ml^−1^ in kinetic buffer (phosphate-buffered saline with 0.1% bovine serum albumin and 0.02% Tween 20) and loaded onto NTA Biosensors by dipping the biosensors into a 96-well plate (Greiner 655209) with 200 µl DARPin per well for 5 min. The biosensors were then dipped into fresh kinetic buffer to establish a baseline (3 min) and subsequently dipped into BARD1 (10 µg ml^−1^) for 5 min (association step), and were then transferred into fresh buffer for 5 min (dissociation step). Each experiment was doubly reference-subtracted using biosensors with zero analyte (BARD1) or that were not loaded with DARPins. Lead candidates were confirmed to bind BARD1 by adding a threefold molar excess of the DARPin to BARD1 and injecting the protein mixture onto an analytical SEC70 column (Bio-Rad Laboratories) equilibrated in 20 m*M* Tris pH 7.5, 150 m*M* NaCl. Fractions were collected and samples from the elution peaks were electrophoresed on SDS–PAGE to identify the protein constituents.

#### 2.1.2. Design, expression and purification of the imaging scaffolds

Anti-BARD1 DARPin sequences identified by yeast display were genetically fused to a tetrahedral nanocage via helical extension with the N-terminus of the DARPin sequence fused to the C-terminus of the cage component (Liu *et al.*, 2018 ▸). Stabilizing mutations (Castells-Graells *et al.*, 2023 ▸) were incorporated to rigidify the trimer interface. DNA sequences were synthesized (Twist Bioscience) and incorporated into bacterial expression vectors: pSAM (Liu *et al.*, 2018 ▸) for subunit A and pET-22b for the subunit B-DARPin fusion.

The plasmids containing both components of the imaging scaffold were co-transformed into *E. coli* BL21-Gold (DE3) cells and the expression and solubility of the two cage components were evaluated at 18°C and 37°C. Designs in which both components were solubly expressed and could be affinity-purified using Ni–NTA beads were chosen for large-scale purification. The imaging scaffolds were grown in 1 l lysogeny broth supplemented with ampicillin and kanamycin to an OD_600_ of ∼0.6 and protein expression was induced with 0.5 m*M* IPTG. The proteins were expressed at 18°C overnight (∼18 h) and harvested by centrifugation. The cell pellets were resuspended in buffer *D* (50 m*M* Tris pH 8.0, 300 m*M* NaCl, 20 m*M* imidazole) and lysed using the same conditions as for SUMO-BARD1, but the protein was purified by affinity chromatography using a gravity column and buffer *E* (50 m*M* Tris pH 8.0, 300 m*M* NaCl, 500 m*M* imidazole) as the elution buffer. Fractions were assessed with SDS–PAGE and those containing both scaffold components were concentrated using a 100 kDa Amicon Ultra-15 concentrator (Millipore Sigma) and further purified by size-exclusion chromatography using a 16/600 Superose 6 column (Cytiva) equilibrated with 20 m*M* Tris pH 8.0, 100 m*M* NaCl. Peak fractions were analyzed by SDS–PAGE and fractions containing both components were pooled and concentrated using a 100 kDa Amicon Ultra-15 concentrator; the purified protein was stored at 4°C pending subsequent X-ray and electron-microscopy experiments.

For scaffold analysis via negative-stain electron microscopy, a 5 µl sample of concentrated protein adjusted to ∼50 µg ml^−1^ was applied onto a glow-discharged Formvar/Carbon 300 mesh (Ted Pella Inc.) for 1 min and blotted to remove any excess liquid. After blotting, the grid was washed three times with sterile Milli-Q water before being stained with a 2% uranyl acetate solution for 1 min. Micrographs were taken on Tecnai T12 and Talos F200C electron microscopes. Negative-stain micrographs were converted to .MRC format and imported into *cryoSPARC* for processing. Micrographs were CTF-corrected using patch CTF correction and ∼3000 particles were manually picked for further analysis. Two rounds of 2D classification resulted in rough averages that were used to assess scaffold assembly. The best 2D classes containing roughly 2000 particles were used to create a low-resolution *ab initio* 3D map with *T* symmetry enforced into which the X-ray structure was docked.

### 2.2. Crystallization

Crystallization screening of BARD1-specific imaging scaffolds using the hanging-drop vapor-diffusion method was conducted at the UCLA–DOE Crystallization Core. Imaging scaffolds (16 mg ml^−1^) and BARD1 (3 mg ml^−1^) were mixed in a 1:1(*v*:*v*) ratio and five 96-well screens were set up using 1:1, 2:1 and 1:2 ratios of protein to reservoir solution (final drop volume of 210 nl) for each condition using a TTP Labtech Mosquito. The screens were incubated at room temperature (∼20°C). Crystals of the DARP3 scaffold were grown by mixing protein solution in a 1:1 ratio with reservoir solution [JCSG+ condition D11: 0.14 *M* calcium chloride, 0.07 *M* sodium acetate pH 4.6, 14%(*v*/*v*) 2-propanol, 30%(*v*/*v*) glycerol]. Prismatic crystals (approximately 70 µm thick) appeared after nine days and were mounted in loops, flash-cooled in liquid nitrogen and stored in liquid nitrogen until data collection.

### 2.3. Data collection and processing

X-ray diffraction data were collected on the microfocus beamline 17-ID-2 at National Synchrotron Light Source II (NSLS-II) located at Brookhaven National Laboratory. Data collection took place at a temperature of 100 K with 0.2° oscillation (1800 frames collected) and an X-ray wavelength of 0.9793 Å. Diffraction data were indexed, integrated, scaled and merged using *XDS* and *XSCALE* (Kabsch, 2010 ▸). Data-collection statistics are reported in Table 1 ▸.

### 2.4. Structure solution and refinement

The structure was solved by molecular replacement using *Phaser* (McCoy *et al.*, 2007 ▸) and a search model consisting of subunit B lacking the DARPin domain (PDB entry 5cy5; Cannon *et al.*, 2020 ▸). The molecular-replacement solution was unambiguous, exhibiting a high positive log-likelihood gain (LLG) of 2533. Difference maps revealed positive residual density for the DARPin domains. A second round of molecular replacement, keeping the cage core fixed, was performed searching for three copies of the DARPin domain using a GFP-specific DARPin (PDB entry 5ma6; 77% sequence identity to BARD1-specific DARPin; Hansen *et al.*, 2017 ▸) as the search model. The molecular-replacement solution further improved the atomic model, as shown by an increase in the LLG to 3348 and a decrease in the *R* factors (*R*_work_ = 0.299, *R*_free_ = 0.327). Manual model building was performed using the graphics program *Coot* (Emsley *et al.*, 2010 ▸). Atomic refinement was performed with *Phenix* (Liebschner *et al.*, 2019 ▸). To minimize overfitting to the 3.8 Å resolution data, noncrystallographic symmetry restraints and conformational restraints to a reference model consisting of PDB entries 8g3k (cage core cryoEM structure at 2.2 Å resolution; Castells-Graells *et al.*, 2023 ▸) and 5ma6 (GFP-specific DARPin cryoEM structure at 2.3 Å resolution) were used. No residual density was observed near the DARPin cargo-binding loops, indicating that BARD1 was not bound in this crystal form. The final atomic refinement statistics are reported in Table 1 ▸. Structure illustrations were created using *PyMOL* (version 1.2r3pre; Schrödinger).

## 3. Results

### 3.1. Selection and characterization of DARPins against BARD1

**Figure 1:**
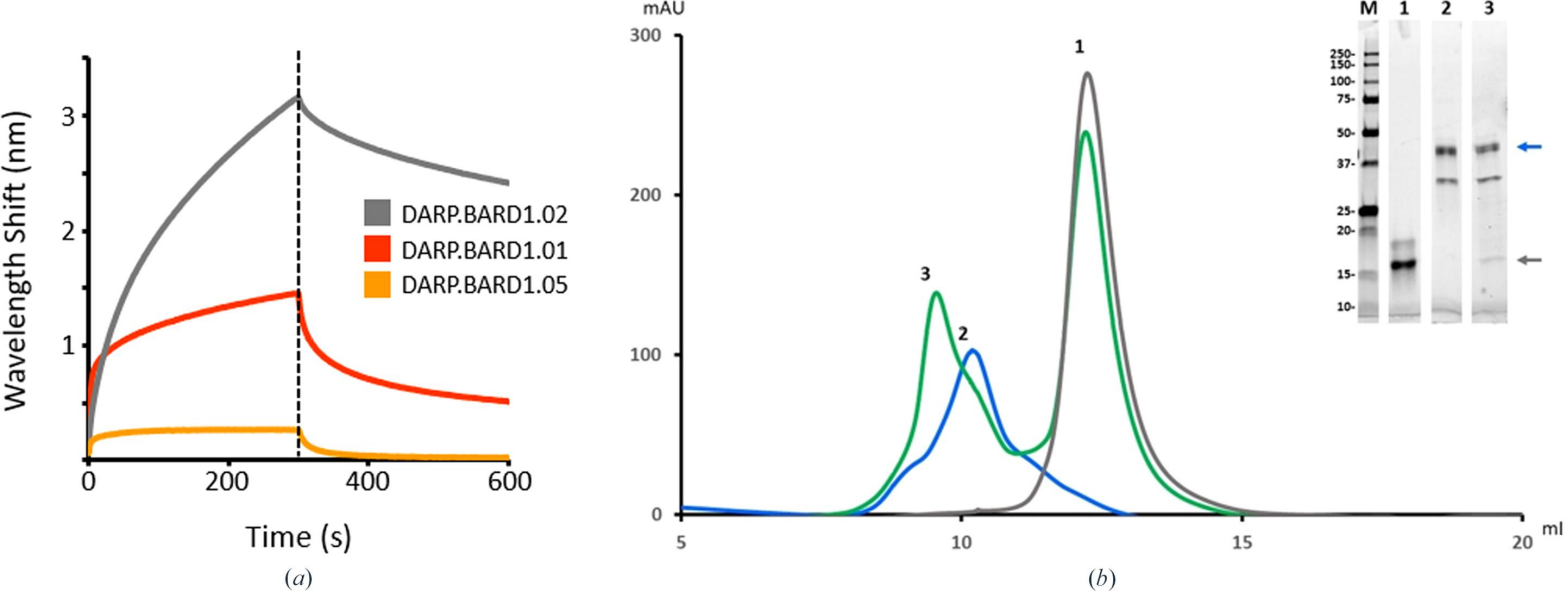
Verification of DARPin–BARD1 binding by biolayer interferometry (BLI) and analytical size-exclusion chromatography (AnSEC). (*a*) Putative anti-BARD1 DARPin molecules were screened for BARD1 binding by loading His-tagged DARPins onto NTA Biosensors and incubating with BARD1 for 5 min and then with analyte-free buffer for 5 min. The large wavelength shifts for DARP.BARD1.01 and DARP.BARD1.02 are indicative of strong antigen binding. (*b*) The size-exclusion profile shows that a DARPin–BARD1 mixture (peak 3) has an altered retention time relative to BARD1 (peak 2) or the anti-BARD1 DARPin (DARP.BARD1.02; peak 1) alone. Inset: SDS–PAGE analysis of the peaks from AnSEC purification. Lane *M*, broad-range molecular-weight marker; lanes 1, 2 and 3 correspond to peaks 1, 2 and 3, respectively. The blue and gray arrows indicate the positions of BARD1 and DARP.BARD1.02 DARPin, respectively.

BARD1 (BRCA1-associated RING domain protein 1) is an important oncogenic protein that forms a heterodimeric complex with BRCA1 (breast cancer gene 1); the complex has E3 ubiquitin activity associated with DNA damage repair and tumor suppression (Brzovic *et al.*, 2001 ▸; Ruffner *et al.*, 2001 ▸; Wu *et al.*, 1996 ▸), and mutations in both BRCA1 and BARD1 are associated with breast, ovarian and pancreatic cancers (De Brakeleer *et al.*, 2016 ▸; Foulkes, 2008 ▸). A yeast DARPin display system was used to generate DARPins against the ligand-binding C-terminal BRCT and ankyrin domain of BARD1 (Watters *et al.*, 2020 ▸). After magnetic- and fluorescence-activated cell sorting, DARPin sequences were isolated from the enriched cell population by PCR and the sequence abundance and diversity were determined by next-generation sequencing (NGS) of PCR amplicons. The ten most abundant sequences ranged between 0.75% to 12% of the total number of sequences (353 K) obtained from NGS sequencing. Five of these sequences were cloned into bacterial expression vectors and were subsequently expressed and purified by affinity chromatography. Interaction with BARD1 was confirmed by biolayer interferometry and by analytical size-exclusion chromatography and SDS–PAGE analysis (Fig. 1 ▸).

### 3.2. Design of the imaging scaffold and biochemical characterization

The helical N-termini of the evolved anti-BARD1 DARPins were genetically fused to the helical C-terminus of the B subunit of a two-component tetrahedral protein nanocage (Cannon *et al.*, 2020 ▸) using recently described stabilizing ‘staple’ mutations at the subunit B trimer interface (Castells-Graells *et al.*, 2023 ▸); subunit A of the tetrahedral assembly is invariant and is the same for all designs. A total of three subunit B-DARPin fusion constructs were made. Together, both components co-assemble into a discrete particle that obeys tetrahedral symmetry and contains 12 copies of the DARPin-fusion subunit and 12 copies of the nonfusion component (four sets of each trimeric protein). The total assembly has a predicted mass of ∼660 kDa and a diameter of approximately 19 nm.

**Figure 2:**
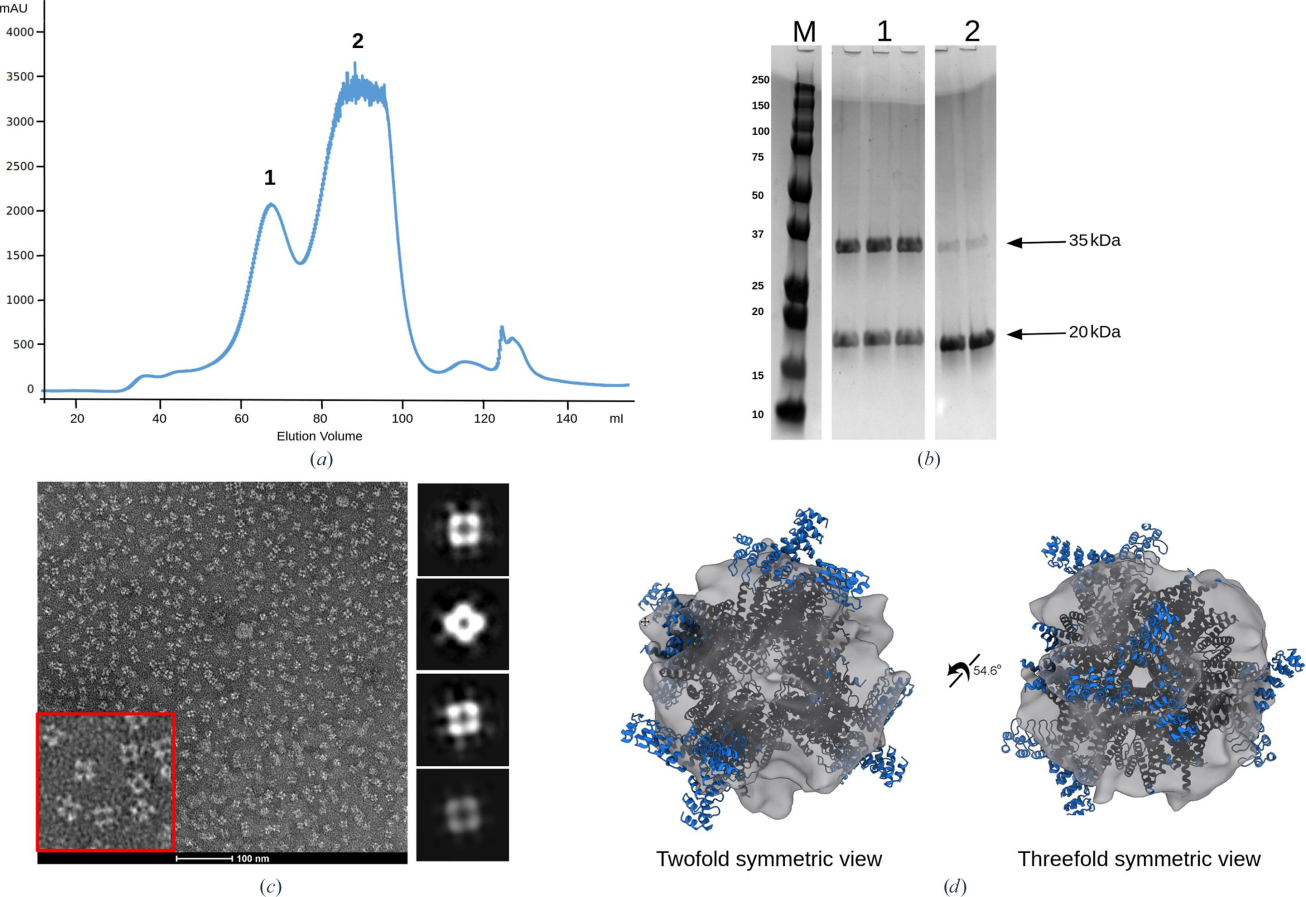
Validation of scaffold assembly. (*a*) The size-exclusion profile shows a peak corresponding to an assembled scaffold (peak 1) and is able to be separated from the unassembled or partially assembled cage components (peak 2). (*b*) SDS–PAGE analysis of the peak fractions shows the presence of both scaffold components at their correct molecular weights, denoted by black arrows. (*c*) Higher order assembly was verified by negative-stain electron microscopy identifying particles of the proper size and symmetry. An enlarged view of the micrograph (red box) shows particles with an estimated diameter of approximately 19 nm, matching the dimensions of the X-ray structure of the DARP3 scaffold (Fig. 3 ▸; PDB entry 8v9o). The particles had a tendency for a preferred orientation along the twofold viewing axis. To the right of the micrograph are rough 2D averages processed from negative-stain data. (*d*) 2D classes were used to generate coarse *ab initio* 3D models.

The plasmids containing the two subunits were co-transformed into *E. coli* and the protein cages were expressed and purified by affinity and size-exclusion chromatography (SEC). Of the three designs that were investigated, only one, DARP3, formed a soluble assembly as assessed by analytical SEC (Fig. 2 ▸ *a*) and SDS–PAGE (Fig. 2 ▸ *b*). Negative-stain electron-microscopy analysis (Figs. 2 ▸ *c* and 2 ▸ *d*) showed particles with the expected tetrahedral geometry and a size of approximately 19 nm, with a preferred orientation displaying its twofold axis of symmetry.

### 3.3. Protein crystallization and structure determination

The DARP3 assembly was subjected to crystallization screening in apo and ligand-bound states. In mixing studies, it was determined that the DARP3 assembly could tolerate only four BARD1 molecules per cage, with amounts of BARD1 in stoichiometric ratios above four cargo molecules per cage (or one BARD1 per DARPin trimer at each vertex) resulting in immediate and severe aggregation, as indicated by an increase in the opacity of the solution upon mixing; this suggests some degree of steric clashing between BARD1 proteins at cage vertices when more than one BARD1 is bound to a DARPin trimer. As a result, the sample was set up with a 1:3 ratio of cargo:DARPin trimer for the ligand-bound state.

**Figure 3:**
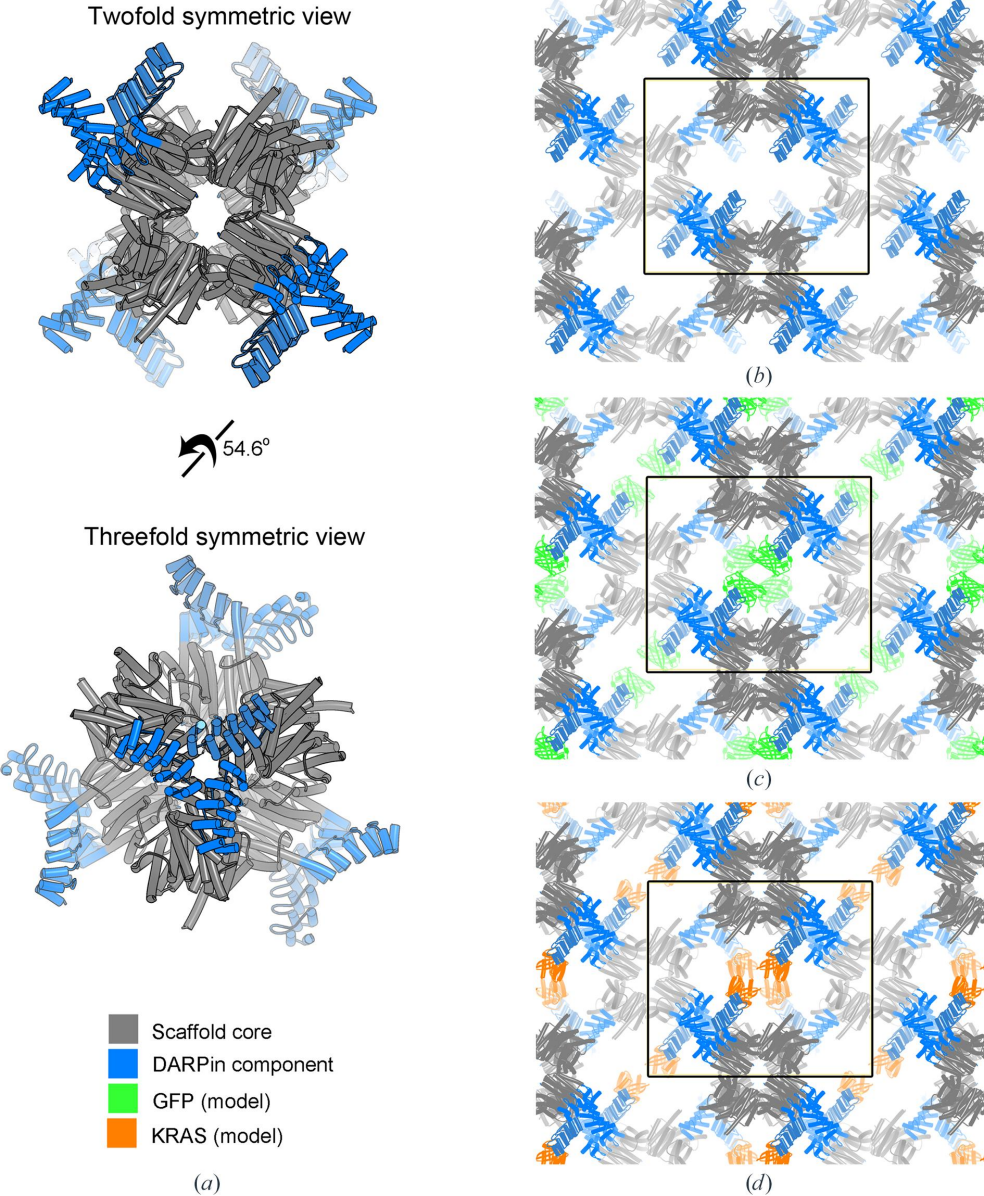
Structure of the DARP3 scaffold and its crystal packing. (*a*) Views of the fully assembled 24-subunit DARP3 scaffold along the twofold (top) and threefold (bottom) axes of symmetry. The crystal structure closely resembles the structure of the KRAS-binding DARPin scaffold (Castells-Graells *et al.*, 2023 ▸). The asymmetric unit consists of a trimer of the DARPin-fused component and the unfused native cage component. (*b*) Crystal packing of the DARP3 assembly. A large solvent channel is present between four copies of the DARP3 scaffold. One DARPin from each scaffold points into the cavity, allowing cargo binding at one of the available DARPins. (*c*) A model illustrating that GFP molecules could theoretically fit into the solvent channel without steric clashes when bound to one of the three DARPin modules. (*d*) A model illustrating that the same is true for KRAS. The black outline denotes the unit cell.

No crystals were found in the crystallization screens for the apo DARP3 assembly; however, crystals in space group *I*222 that diffracted to 3.81 Å resolution were identified in one condition in the screens of the BARD1–DARP3 assembly. The structure was solved by molecular replacement using a single component of the cage (subunit B) and an isolated DARPin molecule as search models. Three copies of subunit A were subsequently fitted to the electron density manually in *Coot* (Emsley *et al.*, 2010 ▸). There was no electron density for the BARD1 cargo protein, indicating that we had crystallized and solved the structure of the apo state of our scaffold. The asymmetric unit contains three copies of subunit A (chains *A*–*C* in the PDB file) and three copies of the subunit B-DARPin fusion (chains *D*–*F* in the PDB file), with the tetrahedral assembly generated via symmetry operations (Fig. 3 ▸ *a*). The structure of the core assembly was first crystallized without DARPin fusions (T33-51H; Cannon *et al.*, 2020 ▸) and there is excellent agreement between the structures of the conserved cage core chains, with an average r.m.s.d. of 0.47 ± 0.03 Å for the superposition of 141 C^α^ atoms of chains *D*–*F* of the DARP3 assembly with chain *B* of the T33-51H assembly; a structure-based superposition, using the *Coot SSM* tool, of chains *A*–*C* of the DARP3 assembly with chain *A* of T33-51H had an r.m.s.d. of 0.36 Å for all three comparisons, with the alignment of 137, 134 and 136 amino acids for DARP3 chains *A*, *B* and *C*, respectively.

The DARP3 assembly crystals have a very high solvent content of 71.47% and a Matthews coefficient of 4.31 Å^3^ Da^−1^. As a result, the lattice has large solvent-filled channels with an approximate cross section of 120 × 180 Å that are periodically restricted by the protrusion of the DARPin moiety of chain *E* into the solvent channel (Fig. 3 ▸ *b*). The DARPin moieties of chains *D* and *F* are involved in mediating crystal contacts in the crystal lattice and thus are unavailable for cargo binding. The substrate-binding face of the chain *E* DARPin is oriented such that substrate binding is possible without creating steric clashes with other components of the lattice. Superposition of the anti-GFP DARPin in the GFP-bound state (Hansen *et al.*, 2017 ▸) onto the anti-BARD1 DARPin in our structure (PDB entry 5ma6, chain *B* residues Lys16–Ala168; DARP3 assembly, PDB entry 8v9o, chain *E* residues Lys169–Ala321) gives an r.m.s.d. of 0.57 Å for the superposition of 153 C^α^ atoms with a sequence identity of 77% and supports the ability of the lattice to support cargo binding, as the GFP barrel, with dimensions of 24 × 42 Å (Ormö *et al.*, 1996 ▸), is oriented in such a way that it does not interfere with the cage core structure (Fig. 3 ▸ *c*). Likewise, the superposition of the structure of the anti-KRAS DARPin bound to KRAS (Guillard *et al.*, 2017 ▸; PDB entry 5o2s) onto DARP3 chain *E* (Fig. 3 ▸ *d*; r.m.s.d. of 0.96 Å for the superposition of 155 C^α^ atoms with a sequence identity of 75.3%) also shows that the binding of a small globular protein cargo within the solvent channel is also possible without physically clashing with the cage core components.

## 4. Discussion

We sought to validate our newly developed DARPin display system (Morselli *et al.*, 2024 ▸) and to use the selected DARPins in conjunction with our suite of designed protein cages to structurally characterize an important cancer-related protein, BARD1. Using yeast display, we identified a number of candidate anti-BARD1 DARPins, and four of these were found, via analytical size-exclusion chromatography, to form stable complexes with BARD1. Three of these candidate DARPins were fused to our improved imaging scaffold using an established protein-fusion strategy (Castells-Graells *et al.*, 2023 ▸), and one of the DARPin-cage fusions was expressed and purified to high yields. SEC and SDS–PAGE analysis showed that the cage fusion eluted as a high-molecular-weight species that contained both subunits in a roughly 1:1 stoichiometric ratio. Negative-stain EM analysis confirmed that we had successfully purified a homogeneous assembly of the expected size and shape.

The primary objective of our protein cage-design projects has been to design imaging scaffolds for the structural characterization of small proteins by cryo-EM. If the designed cage and cargo proteins are available in sufficient quantities, we have also pursued structural characterization of our designs, in apo and ligand-bound forms, by X-ray crystallography. In this project, a single design was expressed in quantities sufficient for crystallization screening. Interestingly, during solution binding studies it was observed that rapid aggregation would occur when the cargo and cage were mixed in ratios corresponding to one BARD1 per DARPin binding site. This result is not totally surprising given that the BARD1 construct used in this study consists of two domains that adopt an extended structure (Dai *et al.*, 2021 ▸) and that the orientation of BARD1 binding to the DARPin is unknown. We hypothesize that this elongated structure may be positioned such that a substantial part of the BARD1 cargo crosses the threefold axis and causes steric clashes with symmetrically related cargo copies. This, compounded with the high affinities that DARPins possess for their cognate ligands, is likely to lead to rapid association between the two, causing cage dissociation and aggregation of dissociated cage subunits. We believe that this aggregation will not occur once the scaffold is locked into the crystal lattice and only one DARPin is left available for ligand binding. In the crystallization trials in this study we loaded the cage with cargo at a 1:1 ratio of ligand to trimeric DARPin binding site to avoid scaffold dissociation.

The DARP3 scaffold with BARD1 cargo produced multiple prismatic crystals of approximately 70 µm in length which diffracted to 3.81 Å resolution. While we have determined structures of similar DARPin-displaying scaffolds by electron microscopy, this is the first instance in which we have determined the crystal structure of a designed cage with cargo-binding DARPin fusions. Unfortunately, the structure is of the apo cage, with no electron density seen for the BARD1 cargo. The most likely explanation for ligand dissociation is the composition of the crystallization solution, which has a low pH (0.07 *M* sodium acetate pH 4.6) and contains a not insignificant concentration of a nonpolar solution (14% 2-propanol) which may interfere with protein–protein interactions and/or protein solubility.

Protein-design efforts focused on creating self-assembling protein cages have been an active area of research since the early 2000s (Padilla *et al.*, 2001 ▸), and a significant number of designed cages have been crystallized and their structures determined (Table 2 ▸). The resolution of crystal structures of protein cages ranges from 2.1 to 7.08 Å, with an average resolution of 3.62 ± 1.68 Å and a median resolution of 3.5 Å for this set of 15 structures including the DARP3 scaffold from this study, which is a derivative of T33-51H (PDB entry 5cy5); if the current structure is excluded the set of cage structures has an average resolution of 3.61 ± 1.34 Å with a median resolution of 3.45 Å. The resolution of the current structure (3.81 Å) is similar to that of the naked T33-51H cage (3.5 Å) and to the median resolution for crystallized protein cages. Higher resolution may be possible through optimization of our existing crystallization conditions or by finding alternative crystal forms via additional crystallization screening. This particular cage assembly has already benefited from strategically engineering staple mutations that stabilize the DARPin near the point of helical extension from the scaffold core (Castells-Graells *et al.*, 2023 ▸) and this new structure will facilitate ongoing protein engineering to further rigidify the scaffold for high-resolution structural studies.

During processing and refinement, it was noted that the crystal contained a high solvent content (71.5%), resulting in large solvent-filled channels throughout the crystal. This agrees with our experience that proteins with high symmetry tend to have fairly high solvent content as they require fewer unique contacts to generate the lattice. Interestingly, one of the three DARPins present at a cage vertex is positioned within the channel formed by the lattice such that it is available for cargo binding. The other two DARPins (chains *D* and *F*) present on the vertex are involved in mediating crystal contacts with adjacent tetrahedral assemblies. With the exception of a single hydrogen bond (2.88 Å; between the carbonyl O atom of Leu167 in chain *C* and the CZ2 atom of Trp209 in chain *F*), the variable cargo-binding surfaces of the DARPins (chains *D* and *F*) are not involved in lattice contacts, and protein–protein interactions occur through conserved invariant residues in the DARPin moieties.

The large solvent channels suggest the possibility that the cage crystals could be soaked with protein substrates which could bind to the free DARPin-binding sites, similar to techniques in which crystals are soaked in solutions of small ligands, allowing cargo-protein structures to be determined. This would be a valuable addition to the structural biologist’s toolbox as an additional salvage pathway through which to determine the crystal structures of proteins that are recalcitrant to crystallization. There are a number of possible complicating factors: the solvent channels may not be large enough to allow proteins to freely diffuse throughout the lattice in the same way that a small molecule can, or penetration of the protein ligand may be incomplete, leading to outer shell DARPin occupancy but leaving the innermost lattice DARPins in their apo state. However, there is a significant upside in that the ligand-binding loops from other DARPins could be grafted on the DARP3 scaffold, allowing easy soaking experiments and structure solution via molecular replacement. These ideas await future studies.

## Supplementary Material

PDB reference: tetrahedral nanocage cage component fused to anti-BARD1 DARPin, 8v9o

## Figures and Tables

**Table 1:** Data-collection and refinement statistics for DARP3 Values in parentheses are for the highest resolution shell.

Data collection	
Beamline	17-ID-2, NSLS-II
Space group	*I*222
Resolution (Å)	3.81 (3.91–3.81)
*a*, *b*, *c* (Å)	128.0, 195.6, 228.4
α, β, γ (°)	90, 90, 90
Measured reflections	191827 (12243)
Unique reflections	28155 (1980)
Completeness (%)	98.9 (96.7)
Multiplicity	6.8 (6.2)
*R*_merge_	0.129 (2.05)
CC_1/2_ (%)	99.9 (48.6)
〈*I*/σ(*I*)〉	11.1 (1.1)
Refinement	
*R*_work_/*R*_free_	0.188/0.225
R.m.s.d., bond lengths (Å)	0.003
R.m.s.d., angles (°)	0.6
No. of protein atoms	10638
No. of water atoms	0
No. of other solvent atoms	1
Average *B* factor, protein (Å^2^)	190
Average *B* factor, water (Å^2^)	N/A
Average *B* factor, other solvent (Å^2^)	159
PDB code	8v9o

**Table 2:** Structures of designed protein cages solved by X-ray crystallography

Protein cage	Symmetry	Resolution (Å)	PDB code	Reference
DARP3	Tetrahedral	3.81	8v9o	This study
T33-51H	Tetrahedral	3.4	5cy5	Cannon *et al.* (2020 ▸)
I52-32	Icosahedral	3.5	5im4	Bale *et al.* (2016 ▸)
I53-40	Icosahedral	3.7	5im5	Bale *et al.* (2016 ▸)
I32-28	Icosahedral	5.59	5im6	Bale *et al.* (2016 ▸)
13 nm cpPduA	Icosahedral	2.51	5hpn	Jorda *et al.* (2016 ▸)
16 nm protein cage	Tetrahedral	4.19	4qes	Lai *et al.* (2016 ▸)
Cube-shaped cage	Octahedral	7.08	4qcc	Lai *et al.* (2014 ▸)
T32-28	Tetrahedral	4.50	4nwn	King *et al.* (2014 ▸)
T33-15	Tetrahedral	2.80	4nwo	King *et al.* (2014 ▸)
T33-21	Tetrahedral	2.10	4nwp	King *et al.* (2014 ▸)
T33-28	Tetrahedral	3.50	4nwr	King *et al.* (2014 ▸)
16 nm cage	Tetrahedral	3.0	3vdx	Lai *et al.* (2012 ▸)
T3-10	Tetrahedral	2.25	4egg	King *et al.* (2012 ▸)
O3-33	Octahedral	2.35	3vcd	King *et al.* (2012 ▸)
